# FULL DOSE CHALLENGE OF MODERATE, SEVERE, AND UNKNOWN BETA-LACTAM ALLERGIES IN THE EMERGENCY DEPARTMENT

**DOI:** 10.51894/001c.122800

**Published:** 2024-08-30

**Authors:** Stephanie Coallier, Reid Mitchell, Adam M. Anderson, Lisa E. Dumkow, Lauren M. Wolf

**Affiliations:** 1 Trinity Health-Muskegon

## O4

### BACKGROUND

Penicillin is the most commonly reported drug class allergy. Reported penicillin allergies often lead to avoidance of both penicillin and beta-lactam antibiotics due to fear of cross-reactivity. Cross-reactivity may be primarily driven by the molecule’s R1 side chain, rather than the beta-lactam ring, supporting challenging documented allergies with antibiotics with dissimilar side chains. Challenging mild allergies with full dose beta-lactam antibiotics has arisen as a practical approach to identify the safe use of beta-lactam antibiotics, however, no current data exists evaluating full dose beta-lactam allergy challenges in ED patients with moderate, severe, or unknown beta-lactam allergies.

### OBJECTIVE

This study aims to assess the outcome of challenging documented moderate, severe, or unknown beta-lactam allergies with full-dose administration of a beta-lactam antibiotic in Emergency Department (ED) patients admitted for acute bacterial infection.

### METHODS

A single center, retrospective, descriptive study of adult patients challenged with full dose beta-lactam in the ED from January 2021 to December 2022 was conducted. Included patients had at least one documented moderate, severe, or unknown beta-lactam allergy in the electronic medical record without documentation of prior tolerance. Patient demographics, prior beta-lactam antibiotic reaction, beta-lactam administered in the ED, inpatient beta-lactam continuation, adverse drug reactions, and updates to allergy profiles were collected. Descriptive statistics for data analysis were performed using SPSS version 22.

### RESULTS

Of the 184 ED encounters with full dose beta-lactam challenges, 5 (2.7%) of the patients with documented moderate, severe, or unknown beta-lactam allergies experienced an allergic reaction after the challenge; 1 (0.5%) patient had an allergic reaction in the ED, the remaining 4 (2.2%) occurred after admission. No anaphylactic reactions occurred. All allergic reactions were limited to mild rash or itching. Most patients (98.9%) were challenged with a cephalosporin. The beta-lactam administered in the ED was continued in 86.4% of cases, and the allergy profile was updated for future utilization in 73.4% of patients.

### CONCLUSIONS

This study suggests that full dose challenge of moderate, severe, or unknown beta-lactam allergies can be safely accomplished in the ED. This approach avoids unnecessary penicillin allergy skin testing and reduces utilization of suboptimal alternative antibiotic regimens.

